# A Design Strategy for Surface Nanostructures to Realize Sensitive Refractive-Index Optical Sensors

**DOI:** 10.3390/nano13243081

**Published:** 2023-12-05

**Authors:** Masanobu Iwanaga

**Affiliations:** Research Center for Electronic and Optical Materials, National Institute for Materials Science (NIMS), 1-1 Namiki, Tsukuba 305-0044, Japan; iwanaga.masanobu@nims.go.jp

**Keywords:** optical sensors, refractive index, label-free, surface nanostructures, surface lattice, metasurfaces, plasmons, dielectric resonances

## Abstract

Refractive-index optical sensors have been extensively studied. Originally, they were surface plasmon resonance sensors using only a flat gold film. Currently, to develop practically useful label-free optical sensors, numerous proposals for refractive index sensors have been made using various nanostructures composed of metals and dielectrics. In this study, we explored a rational design strategy for sensors using surface nanostructures comprising metals or dielectrics. Optical responses, such as reflection and transmission, and resonant electromagnetic fields were computed using a numerical method of rigorous coupled-wave analysis combined with a scattering-matrix algorithm. As a result, good performance that almost reached the physical limit was achieved using a plasmonic surface lattice structure. Furthermore, to precisely trace the refractive-index change, a scheme using two physical quantities, resonant wavelength and reflection amplitude, was found to be valid for a 2D silicon metasurface.

## 1. Introduction

Optical sensors are being developed for diverse targets, from gaseous molecules to large biomolecules such as antibodies and DNA. A significant feature of optical sensors is their direct contact with target molecules and the magnification of detection signals owing to their resonantly enhancing mechanisms. Considerable efforts have been devoted to the development of practical optical sensors for various applications.

Refractive-index optical sensors are label-free sensors that enable evaluation of the refractive index of the medium with which the optical sensors are in contact. Originally, refractive-index sensors were validated using surface plasmon resonances (SPRs) in the visible range [1]. SPRs are typically observed using a gold thin film deposited on a glass prism at deep oblique p-polarized incidence. In addition to the refractive index of the surrounding medium, SPR sensors can evaluate the mass of molecules immobilized on the outermost surface of gold, which makes them useful for quantitatively analyzing biomolecule reactions involving immobilization and dissociation [2]. Thus, refractive-index sensors have the potential to function as mass analyzers for immobilized molecules. The requirement of an elaborate optical configuration that includes a total-reflection setup is a drawback of SPR sensors. Therefore, the development of refractive-index sensors that can operate in compact and simple optical configurations using surface nanostructures has been explored in the visible and near-infrared ranges [3,4,5,6,7,8,9,10,11,12,13]. From a similar motive, fiber-based refractive-index sensors were studied [14,15,16,17,18]; these sensors have long light propagating paths and high responsivity in the near-infrared range, while the sensing range is mostly limited to a narrow refractive-index range of 1.35–1.40.

Despite the numerous proposals for refractive-index sensors, designing the high-performance sensors in a straightforward manner remains a challenge. Here, we first arranged the design flow and then studied several plasmonic and dielectric surface nanostructures that exhibited explicit responses as refractive-index sensors in the wavelength range of 500–1100 nm, where optical measurements are most cost-effective using silicon photon detectors. In this study, the physical limitations of the refractive-index sensors were clarified. Furthermore, one of the plasmonic sensors was found to exhibit a sensing performance that reached the physical limit. Other surface nanostructures stemming from ideas differing from the nearly ideal plasmonic sensor were also studied to examine their suitability as refractive-index sensors.

**Figure 1:**
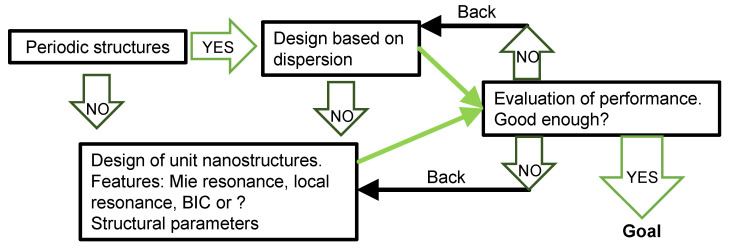
Design flow of optical sensors. The options or key steps are shown in boxes, while the flow is indicated by arrows.

A flow to design the optical sensors is shown in Figure 1. The goal is to realize high-performance practical refractive-index sensors. First, a basic structure, either periodic or non-periodic, is chosen. If periodic structures are chosen, the next step is whether or not to conduct designs based on dispersion: (i) if yes, feasible periodic structures realizing specific dispersions are designed, tested using a numerical simulation method, and evaluated in terms of performance as a refractive-index sensor; (ii) if no, nanostructures are designed based on interest and/or preference by each researcher. From this reason, Mie resonances in metallic nanoparticles [4,5], local resonances in metallic/dielectric nanostructures [3,6,7,8,9,10], and bound states in the continuum (BIC) [11,12,13] have often been studied. Artificial surfaces were designed by assembling the nanostructures on a substrate; subsequently, the surface structures were tested using the simulation method, and their performance was evaluated. These processes continued until an appropriate design was obtained.

Various algorithms have been used to investigate artificial nanophotonic structures. Recently, inverse designs have gained popularity and have been used for metalenses [19,20,21], transmission waveguides [22,23], and metasurfaces [24,25]. Other search methods have been proposed. Nonempirical searches for nanostructures with optical functions have been used to obtain new structures [26]. Genetic algorithms have also been used to search for photonic structures [27,28,29,30,31,32]. However, to our knowledge, refractive-index optical sensors have not yet been designed using these search algorithms. This is partially because multifactors, including constituent materials, should be simultaneously optimized for refractive-index sensors, which complicates the search algorithm and makes its implementation difficult.

## 2. Methods and Material Parameters

In this article, we address periodic nanostructures and employ the rigorous coupled-wave analysis (RCWA) [33], which incorporates a scattering-matrix algorithm [34] to stably compute arbitrary stacked structures. The results of the numerical method that was coded in Fortran 90 and executed using multiparallel implementation acceleration on supercomputers were consistent with experimental data in various cases [35]. The RCWA solves Fourier-transformed Maxwell equations for periodic structures and approximates the exact solutions using the inverse Fourier factorization and truncating the Fourier expansion [33]. To conduct realistic computations, the material parameter, permittivity, were taken from the literature [36,37], and the measured permittivity was used for Au and Si. Among the various metals, Au was chosen because of its high chemical stability. Si was selected because of its high permittivity. The permittivity of SiO_2_ was set to a representative value of 2.1316.

## 3. Results

In this section, concrete designs are numerically studied. 1D and 2D surface-lattice structures are addressed in both plasmonic and all-dielectric cases. The physical limit of refractive-index sensing is explicitly described in Section 3.1.

### 3.1. 1D Plasmonic Surface Lattices

**Figure 2:**
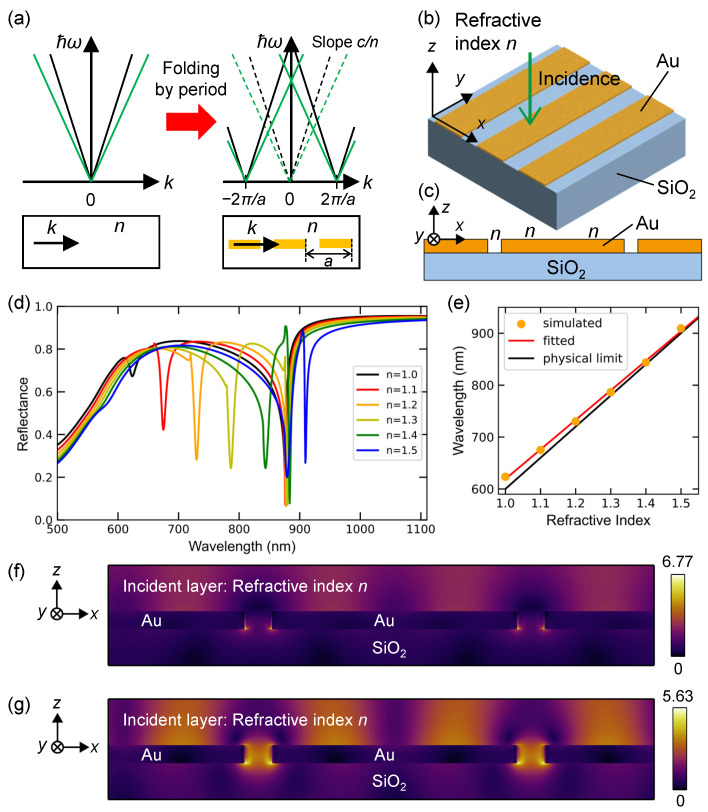
(**a**) Schematics of the dispersions of uniform (**left**) and periodic (**right**) systems on the (k,ℏω) planes. (**b**) 1D plasmonic surface-lattice structure and optical configuration. The periodicity was set to 600 nm. (**c**) Illustration of the xz section of (**b**). The refractive index *n* is shown at typical points. (**d**) Computed reflectance spectra for the refractive index *n* from 1.0 to 1.5 in the incident layer, respectively. (**e**) Refractive-index sensing performance: the physical limit (black line) in Equation (2) and the simulated data (orange dots) selected from (**c**) and fitted by a linear function (red line). (**f**,**g**) Resonant electric field distributions, |E| (xz-section view as shown in (**c**)) at the wavelengths of the reflectance dips in (**c**) for n=1.0 and 1.4, respectively.

The dispersions of light are illustrated on the (k,ℏω) planes in Figure 2a, where *k* and ω denote the wavenumber and angular frequency, respectively. On the left, the light cone (which is the dispersion in uniform media illustrated in the box below) for refractive index n=1.0 is drawn with black lines, while that for n>1 is shown with green lines for comparison. On the right, the 1D lattice structure of periodic length *a* is depicted in the box below, where we assume that the periodic structure (orange) is infinitely long perpendicular to the sheet of paper.

The dispersions in Figure 2a show four lines located inside the light cones (dashed lines). This effect is often referred to as the folding of dispersion into the first Brillouin zone, that is, −π/a<k<π/a. Cross points appear at k=0 owing to the periodic structure. The dispersions at k=0 allows the excitation of optical modes at normal incidence. In the surface lattice, the *k* vector is along the direction of the periodic structure. Although mode coupling at k=0 usually results in small band gaps at the cross points [38], we omit the gaps for simplicity. A line-dispersion equation such that

(1) $$\hbar \omega =\frac{\hbar c}{n}\left(k+\frac{2\pi }{a}\right)$$

is one of the green lines in Figure 2a (right). From Equation (1), we can derive a simple equation between the resonant wavelength λ and refractive index *n* at k=0:

(2) λ=an.

where ω=2πc/λ (*c*: the velocity of light in vacuum). Equation (2) represents the physical limit (or theoretical limit) for the dispersion relation that can be realized in 1D surface lattices. We emphasize that Equation (2) indicates that the ideal refractometric response Δλ/Δn is determined only by the periodic length of the lattice structure, being expressed such that

(3) $$\frac{\Delta \lambda }{\Delta n}=a.$$

The dispersions for 2D surface lattices are more complicated than those for 1D surface lattices, and should be evaluated as photonic bands in slab structures [39]. The slopes of the dispersion curves in the 2D lattices are smaller than those of the light cones, resulting in a smaller refractive-index sensing performance than that indicated by Equation (3).

A 3D illustration of a 1D plasmonic surface lattice is shown in Figure 2b. The optical configuration and spatial xyz coordinates are presented. The incident plane wave comes from the top and travels along the −z direction. Thin Au bars with a thickness of 40 nm are placed periodically along the *x* axis and are infinitely long along the *y* axis. The periodicity along the *x* axis is 600 nm, and the widths of the Au bars and slits are 540 and 60 nm, respectively. The xz-section view is illustrated in Figure 2c.

A series of reflectance spectra under the incident polarization of $E_{\mathrm{in}}\|x$ are shown in Figure 2d, changing the refractive index from 1.0 to 1.5 in the incident layer and slits between the Au bars; the corresponding colors are black, red, orange, yellow, green, and blue, respectively. The slit width was set to 60 nm. An evident response to the refractive index appears as a large shift of the reflectance dips from 623 to 909 nm. We mention that several reflectance dips at approximately 880 nm originate from the first diffraction mode into the SiO_2_ substrate, which is distinct from the mode sensitive to the refractive index. In practice, a wavelength range of approximately 880 nm is unsuitable for refractive-index sensing. We also mention that the reflectance spectra under $E_{\mathrm{in}}\|y$ qualitatively resemble those of the flat Au film and do not exhibit definite resonances, as shown in Figure 2d.

In Figure 2e, the response to the refractive index *n* is compared with the ideal response in Equation (3). The simulated data (orange dots) were collected from the reflectance dips in Figure 2d and fitted using a linear function (red line). The black line represents the response in Equation (2). The response of the 1D plasmonic surface lattice approaches the physical limit, suggesting the realization of an almost ideal response, whereas a small deviation is observed in Figure 2d, which suggests that optical loss in Au degrades the performance. The reflectance-dip wavelength at n=1.0 is 623.5 nm, where Au is more lossy than 843.8 nm for n=1.4 and 909.4 nm for n=1.5 [36]. This results in the deviation from the ideal response shown in Figure 2d. The unit of nm/refractive index unit (RIU) is conventionally used to evaluate refractive index sensors. The 1D plasmonic lattice is estimated to possess 569.1 nm/RIU, which almost realizes the limit of 600 nm/RIU implied by Equation (3). From the dispersion, the resonant mode sensitive to the refractive index is ascribed to the first-order dispersion folding mode, as illustrated in Figure 2a. The folding mode is associated with the diffraction induced at the interface of the incident layer and the 1D lattice; therefore, it can be called the first-order diffraction mode.

Figure 2f,g shows xz-section-view electric-field distributions of |E|, which have two slits in each panel. The incident layer was set to have refractive indices of 1.0 and 1.4, and incident wavelengths were set to 623.8 and 843.8 nm, respectively, in accordance with the reflectance dips in Figure 2d. The color bars indicate the values of |E|, which were normalized by setting the incident $|E_{\mathrm{in}}|=1$. Clearly, the most enhanced fields appear in the slits between the Au bars and exhibit |E| fields that are enhanced several times, thereby probing the refractive index *n*. However, the field enhancement at the slits is not sufficiently strong; therefore, the refractometric response originates primarily from the poriodic structure. The narrow slits contribute to high reflectance, except for the resonant wavelengths, resulting in high-contrast reflectance spectra. Wider slits will reduce the contrast in the reflectance spectra.

### 3.2. 2D Plasmonic Surface Lattices

**Figure 3:**
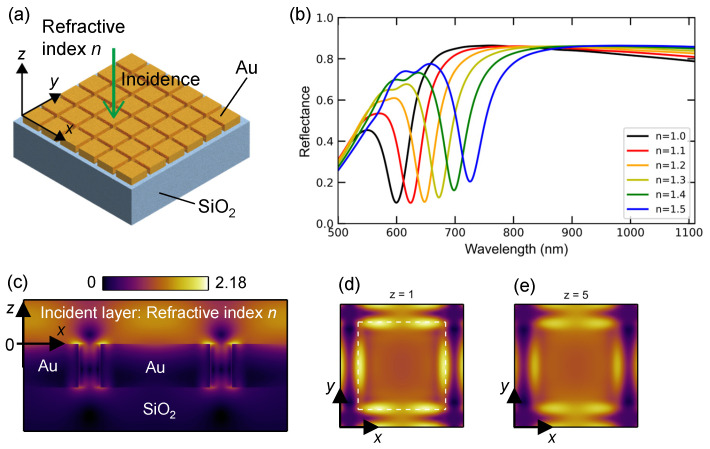
(**a**) Schematic of 2D plasmonic surface lattice on SiO_2_ substrate. The periodicity of the square lattice was set to 300 nm. (**b**) Reflectance spectra dependent on refractive index *n* in the incident layer of 1.0 and 1.5, respectively. (**c**–**e**) Resonant |E| distributions in one xz-section view and two xy-section views, respectively. The position of an Au nanocube is indicated with a dashed white square in (**d**). The scale bar is in common for these three panels.

A 2D plasmonic surface lattice is schematically illustrated in Figure 3a. Assuming a square lattice of Au nanocubes with a periodicity of 300 nm on a transparent SiO_2_ substrate, the optical modes at normal incidence are independent of polarization. The xyz coordinates were set as shown, and the incident plane wave propagated from the top. The refractive index of the incident layer and slits between the Au nanocubes was assumed to be *n*. This nanostructure is based on the idea that (i) 2D lattices generally have complicated photonic modes, which require a shorter periodicity than that of the 1D lattice; (ii) a thick plasmonic lattice sometimes induces deep reflectance modes, and a thickness of 100 nm or greater is preferred; and (iii) a polarization-independent lattice is chosen. Thus, the design was assembled from the tips for the plasmonic nanostructures.

The numerically calculated reflectance spectra at normal incidence are shown in Figure 3b, and the color presentation is similar to that in Figure 2d. The refractive indices of the incident layer and slits between the Au nanocubes ranged from 1.0 to 1.5. The slit width and height of the Au nanocubes were set to 50 and 100 nm, respectively. Then, a series of deep reflectance dips of 0.1–0.2 appears at 600–720 nm. The resonant wavelength of 600 nm for n=1.0 is considered to originate from the second-order folding mode at k=0. Other prominent resonances were suppressed, and consequently, the refractometric response was evident. The sensitivity was estimated to be 252.4 nm/RIU. In this 2D plasmonic surface lattice (or metasurface), the maximum sensitivity is limited to 300 nm/RIU, according to the physical limit in Equation (3). Thus, this 2D plasmonic lattice exhibited high responsivity as a refractive-index sensor. We note that in the 2D plasmonic lattice of 40 nm height and the same periodicity and slit width exhibited shallow reflectance dips, which made them unsuitable for refractive-index sensing.

Next, we examine the resonant mode at the deep reflectance dip from the electric field distributions. Figure 3c shows an xz-section view of |E| distribution; the section crosses the center of the Au nanocube. The position of z=0 was set at the interface between the incident layer and Au lattice. The scale bar indicates the values of |E| for incident $|E_{\mathrm{in}}|\;=1$. The most enhanced electric field appears at the top of the Au nanocubes and near the slits; the value of |E| is approximately twice the maximum, compared to that of the incidence. The xy-section views of |E| distribution are shown in Figure 3d,e at z=1 and 5 nm, respectively, where the *z* positions are close above the top surface of the 2D plasmonic lattice. The position of the Au nanocube in the image is shown with dashed white lines in Figure 3d and is set similarly in Figure 3e. The scale bar is similar to that in Figure 3c.

### 3.3. 1D Silicon Surface Lattices

**Figure 4:**
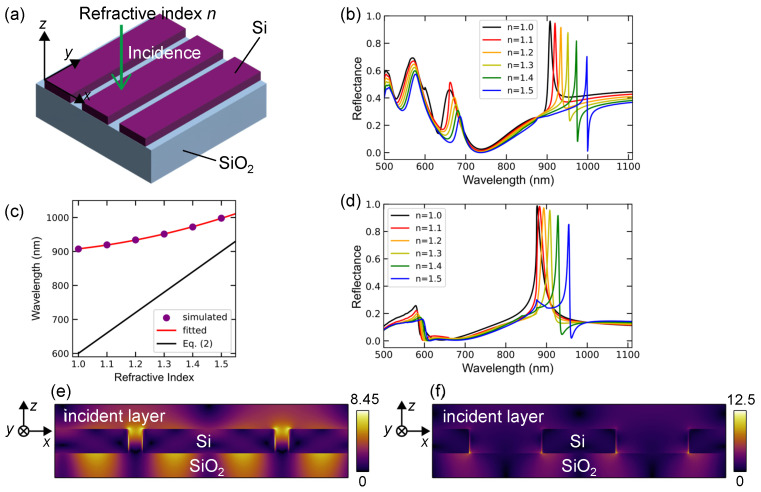
(**a**) Schematic of the 1D Si surface-lattice structure. The periodicity is 600 nm. (**b**) Polarized reflectance spectra of a 1D lattice with a Si bar width of 540 nm for $E_{\mathrm{in}}\|x$. The spectra depend on the refractive index *n* from 1.0 to 1.5 in the incident layer, respectively. (**c**) Sharp peak wavelengths in (**b**) are plotted for the refractive index and fitted using a quadratic function (red curve). Equation (2) is represented by a black line. (**d**) Polarized reflectance spectra of a 1D lattice with a Si width of 300 nm, computed and displayed in a manner similar to (**b**). (**e**,**f**) Resonant |E| distributions, corresponding to the highest reflectance peaks for n=1.0 in (**b**,**d**), respectively. The xz-section views are presented.

A schematic of the 1D Si surface lattice structure is shown with the xys axes in Figure 4a. The periodicity along the *x* axis was set to 600 nm, and the Si bars were infinitely long along the *y* axis. The propagation direction of the incident light is indicated by the green arrow. This 1D Si lattice was designed for comparison with the 1D plasmonic lattice shown in Figure 2.

The computed reflectance spectra of the 1D Si surface lattices with a Si bar width of 540 nm (i.e., slit width of 60 nm) are shown in Figure 4b. The reflective index in the incident layer and the slits between the Si bars varied from 1.0 to 1.5. The color representation is similar to that shown in Figure 2d. The reflectance peaks at 900–1000 nm are plotted in Figure 4c (purple dots) and fitted using a quadratic function (red curve). The line suggested by Equation (2) is also shown for comparison. The deviation of the reflectance peaks from the line is obvious, indicating that the peaks are located at longer wavelengths than the line. The peak wavelengths are close to the diffraction at the interface of the Si lattice and substrate; the diffraction mode appears at 876 (=600×1.46) nm at the normal incidence. The slope, which depends on the refractive index, is quadratic and deviates from a linear response, suggesting that the response to the refractive index is less sensitive.

**Figure A1:**
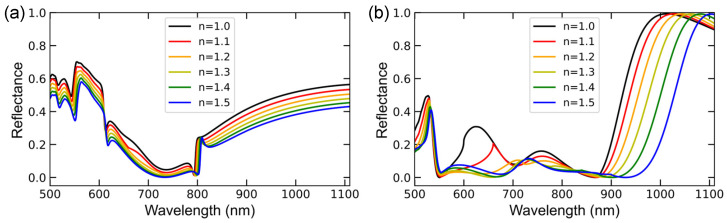
(**a**,**b**) Reflectance spectra of 1D Si surface lattices under $E_{\mathrm{in}}\left|\right|y$, corresponding to Figure 4b,d, respectively.

The computed reflectance spectra of the 1D Si surface lattices with a Si bar width of 300 nm (i.e., slit width of 300 nm) are shown in Figure 4d. Prominent reflectance peaks appear in the wavelength range of 875–950 nm in Figure 4b,d, and the resonant peak shift for a Si-bar width of 540 nm is larger than that for a 300 nm width bar. This suggests that the Si surface nanostructure with narrow slits is more sensitive to the refractive index, probably because the wider Si bars maintain the diffraction mode at the interface of the Si lattice and substrate, which is visualized in the following electric-field distributions. We mention that the 1D Si surface lattices are less sensitive for refractive index under *y* polarization in Figure A1 (Appendix A) than the *x* polarization in Figure 4b,d.

The resonant electric fields are shown in Figure 4e,f, which correspond to the reflectance peak for n=1.0 in Figure 4b,d, respectively. The most enhanced fields appeared inside the slits between the Si bars and near the interface of the Si bars and SiO_2_ substrate. Moreover, periodic field distribution in the SiO_2_ substrate indicates a diffraction mode into the substrate. We note that the values of |E| are larger in the 1D Si lattice than in the 1D plasmonic lattice in Figure 2f,g. Thus, field enhancement is expected in Si surface lattices or metasurfaces. Indeed, the prominent fluorescence enhancement of more than 1000-fold has been achieved in Si metasurfaces [40,41,42,43].

Unlike the 1D plasmonic surface lattice, the 1D Si surface lattice did not function as a refractive-index sensor in the visible range. Furthermore, its response to the refractive index is less sensitive than that of the 1D plasmonic lattice. Designs based on dispersion are most likely difficult for Si surface lattices.

### 3.4. 2D Silicon Surface Lattices

**Figure 5:**
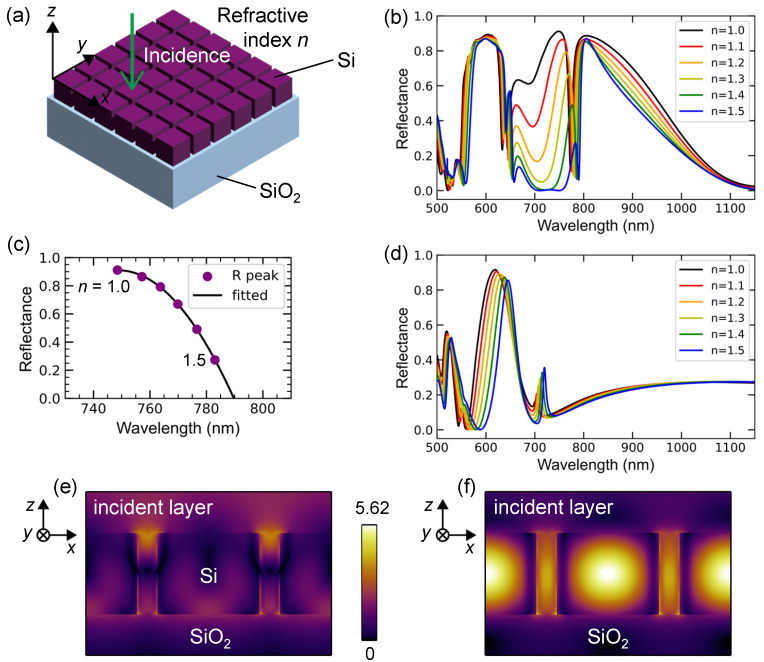
(**a**) 2D Si surface lattice structure. The lattice was square with a periodicity of 300 nm. (**b**) Numerically calculated reflectance spectra of a 2D lattice with a height of 200 nm. (**c**) Shift of the reflectance (R) peak (purple dots) fitted using a quadratic function (black curve). (**e**) Numerically calculated reflectance spectra of a 2D lattice with a height of 100 nm. The spectra in (**b**,**d**) depend on the refractive index *n* from 1.0 to 1.5 in the incident layer and slits between the Si nanocubes. (**e**,**f**) Resonant electric-field distributions, |E|, of the 2D lattice with a height of 200 nm for n=1.0 at an R peak of 748.5 and a R dip of 772.8 nm, respectively, are shown in an xz-section view. The color bar is in common for (**e**,**f**).

A 2D Si surface lattice on an SiO_2_ substrate is schematically illustrated in Figure 5a. The xyz axes and direction of incidence are also shown. The lattice was a square array of Si nanocubes with a periodicity of 300 nm. We can call the lattice metasurface. The idea of designing this Si lattice is partly similar to that of the 2D plasmonic lattice. We aimed to avoid complicated dispersions by setting a short periodicity of 300 nm. However, because dielectric nanostructures such as cubes and spheres exhibit Mie resonances [44,45], it is unclear whether the desired sensing performance can be obtained.

The computed reflectance spectra of a 2D Si lattice with a height of 200 nm are shown in Figure 5b. The refractive indices in the incident layer and slits between the Si nanocubes varied from 1.0 to 1.5. The color representation is similar to that shown in Figure 2d. Distinct spectral changes are observed in the 660–800 nm range. The change in the reflectance peak is indicated by purple dots in Figure 5c, which begins at n=1.0 and decreases to the right as *n* increases. The peak varies both in wavelength and reflectance; therefore, it is plotted on the (wavelength, reflectance) plane and fitted well using a quadratic function (black curve). Thus, quantitative refractive-index sensing is possible by detecting reflectance spectra in the wavelength range of 740–800 nm. Notably, the change in reflectance provides another new dimension in addition to wavelength; that is, quantification such as (wavelength, reflectance) →n allows more precise sensing than that relying only on the wavelength change because the parameter space is extended. We note that a commercial SPR sensor instrument measures changes in reflectancerather than changes in wavelength [2], probably because a change in reflectance is more feasible for precisely attaining a wide range of signal changes.

The reflectance spectra for the 2D Si lattice with a height of 100 nm were also calculated in a manner similar to that in Figure 5b, as shown in Figure 5d. The large spectral changes in Figure 5b disappear, indicating a less sensitive response to the refractive index. This type of sensing has been reported for biomolecular sensing at moderate precision; the spectral shift was less than 3 nm [5]. Thus, more sensitive refractive-index sensors can find applications for specific purposes.

The resonant modes related to the change in the reflectance spectra are shown in Figure 5e,f, where xz-section views across the centers of the nanocubes are presented. A reflectance peak at 748.5 nm and a dip at 772.8 nm for n=1.0 (black curve in Figure 5b) correspond to Figure 5e,f, respectively. The color bar is in common for the two panels, and the values are normalized under the assumption of $|E_{\mathrm{in}}|\;=1$. At the wavelength of the reflection peak, the electric-field distribution of | E| is most enhanced (4.1-fold) at the entrance of the slits between the Si nanocubes, whereas at that of the reflectance dip, the electric fields oscillate inside the Si nanocube and are 5.6-fold enhanced, resulting in effective transmission into the substrate. The electric fields at the wavelength of the reflectance peak are suitable for probing the refractive index in the incident layer and slits.

## 4. Discussion

We numerically studied 1D and 2D plasmonic/all-dielectric surface-lattice structures to identify suitable candidates for refractive-index sensors. The physical limit of the performance of refractive-index sensors was clarified in Equation (3). A simple evaluation using the unit of nm/RIU was found to be insufficient because the limit depends on the periodicity of the structures; a large periodicity structure tends to show a larger response in nm/RIU. Thus, a simple comparison of the values of nm/RIU is meaningless. For a meaningful comparison, we here introduce a factor, realization of limit (RoL), which is defined as

(4) $$\mathrm{RoL}=\frac{\mathrm{shift}\;\;\mathrm{in}\;\;\mathrm{nm}/\mathrm{RIU}}{\mathrm{structural}\;\;\mathrm{periodicity}}\;\;\left(\%\right).$$

In addition to the RoL in Equation (4), other features should be considered. Some surface lattices exhibit a nonlinear response (or wavelength shift), as shown in Figure 4c and Figure 5c. Thus, the shift is not be a unique performance indicator. In practice, reflectance changes can be useful, considering the current commercial SPR instruments. Thus, we compared the surface lattices in terms of multiple factors, as listed in Table 1.

In the case of analysis of refractive-index sensors with small wavelength shifts, the resonant line width should be taken into account [11,12,13]. However, we here focus on the surface nanostructures with large shifts and omit the discussion on the effects of the linewidth.

In Table 1, the highest RoL (≈95%) is obtained for the 1D plasmonic lattice in Figure 2. Although the 1D structure is polarization-dependent, this drawback is overcome by the 2D plasmonic lattice shown in Figure 3. Both plasmonic structures functioned as refractive-index sensors, primarily in the visible range, and exhibited a linear wavelength shift. SPR was tested in terms of refractometric response on flat Au film or nanoparticles (including spheres, triangles, and prisms), and biomolecules were detected in a small shift of a few nm [4].

On flat Au films, SPR can be excited using an optical prism [2]. The dispersion is nonlinear, and the observed wavelength shift becomes nonlinear; consequently, its evaluation using nm/RIU is improper. However, such an inaccurate evaluation was conducted, and an unrealistically large resonance (>10,000 nm/RIU) was claimed in an early stage of the research [47].

In the Si surface lattices in Figure 4 and Figure 5, the resonant modes sensitive to the refractive index show quadratic responses that originate from photonic band structures. In the 2D Si lattice, the reflectance varies significantly under normal incidence, which makes refractive-index sensing feasible, similarly to the commercial SPR instrument. To our knowledge, such a large reflectance change has not been reported for Si surface nanostructures, including Si metasurfaces. Thus, the features of 2D Si lattices can contribute to practical refractive-index sensors in a compact optical setup equipped with only a Si photon detector. The Si metasurfaces reported to data [11,12,13] were designed to work at telecom wavelengths of approximately 1.55 µm. The RoL was lower than those of the plasmonic lattices in Table 1, probably because the BIC associated with the high-quality factor was focused on, and the RoL was not optimized. At present, the compatibility of high-quality-factor BIC and high RoL is unclear.

We refer to a hollow-core fiber-based refractive-index sensor [16] listed in Table 1. A linear-response approximation at a very narrow range of refractive index of 1.43–1.44 can result in a large value of 19014 nm/RIU. However, the fiber-based sensors usually show quadratic refractometric responses, and in general, the linear approximation is invalid. We note that the fiber-based sensors work only at a small range of refractive index, typically 1.35–1.40 [14,15,16,17,18] because the operation principle is transmission through a long optical paths (∼mm) and interference. Thus, the fiber-based sensors are not comparable to the surface nanostructure sensors functioning at refractive index of 1.0–1.5.

Refractive-index sensors are explored in THz ranges. An simulated result [46] is listed in Table 1. In THz ranges, refractive indices of materials are considerably different from those in the visible and near infrared ranges, and tend to be significantly larger. Thus, the sensors in THz ranges are not directly comparable to those studied in this article. In addition, it is to be noted that the detection range is narrow, limited to n=1.34–1.44 [46].

The refractive-index sensors studied here have features in wide-range sensing for the range n=1.0–1.5, large RoL, and dynamical change in wavelength and reflectance. These features are considered to make the application for biomolecule analysis feasible in a similar manner to the current commercial SPR sensors. One benefit of these sensors enables simple and compact optical setup, compared with the SPR sensors, and will lead a portable reflective-index and biomolecule-analyzing sensors for diverse targets.

## 5. Conclusions

Designs for high-performance refractive-index sensors have been explored for plasmonic/all-dielectric surface-lattice platforms. The physical limit of the refractometric response was first revealed, and concrete designs were then studied from the viewpoints of spectroscopy and resonant modes. Consequently, we found that the 1D plasmonic surface lattice is a nearly ideal refractive-index sensor, while the 2D plasmonic lattice is a high-performance sensor independent of incident polarizations. In addition, we showed that the 2D Si surface lattice exhibited a reflectance change for the refractive index, which was similar to a commercial SPR setup. Howver, the Si lattice operated at the normal incidence, whereas the SPR sensors required deep oblique and polarized incidence. Thus, the designs presented here are useful for constructing compact, simple, and cost-effective refractive-index sensing setups, which have potential to serve as portable and handy sensors outside laboratories.

## Figures and Tables

**Table 1:** Comparison of the refractive-index sensors proposed to date. RoL is defined in Equation (4). Dim, NA, MSF, Exp, and Sim denote dimension, not available, metasurface, experiment, and simulation, respectively.

Structure	Dim	RoL (%)	Other Features / Notes	References
Plasmonic lattice	1D	94.9	linear shift at 623–880 nm.	
			569.1 nm/RIU	This study
Plasmonic lattice	2D	84.1	linear shift at 600–720 nm.	
			252.4 nm/RIU	This study
Au thin film	2D	NA	50 nm/RIU. BIACORE instrument	[2]
Au nanoprism	2D	NA	800–850 nm. local SPR of random	
			array. shift less than 10 nm	[4]
Si lattice	1D	NA	quadratic shift at 900–1000 nm	This study
Si lattice	2D	NA	large reflectance change & quadratic	
			shift at 745–790 nm	This study
Si MSF	2D	51.6	Exp around 850 nm. Mie resonance.	
			50 nm height nanodisk	[5]
Si MSF	2D	54.3	1550–1600 nm. Sim 440 nm/RIU.	
			BIC. Asymmetric pair rods.	[11]
Si MSF	2D	69.1	1300–1450 nm. Exp 608 nm/RIU.	
			BIC. 450 nm height pair rods.	[12]
Si MSF	2D	45.9	Exp around 1550 nm. BIC.	
			30 nm nanogap	[13]
Hollow-core	3D	NA	Exp n=1.43–1.44 at 1450–1650 nm.	
fiber			19,014 nm/RIU. length ∼mm	[16]
THz absorber	2D	73.9	Sim n=1.35–1.44 at 8.3–8.9 THz.	
			25.9 µm/RIU. periodicity 35.0 µm	[46]

## Data Availability

Data are available from the author upon reasonable request.

## Abbreviations

The following abbreviations are used without definition in this manuscript:

1D	One-Dimensional
2D	Two-Dimensional
3D	Three-Dimensional
DNA	Deoxyribonucleic Acid

## Appendix A. Optical Responses of 1D Silicon Lattice at Another Polarization

Figure A1 shows the reflectance spectra of the 1D Si lattice shown in Figure 4a. The incident polarization was set as $E_{\mathrm{in}}\left|\right|y$, parallel to the Si bars and perpendicular to the polarization in Figure 4b,d. It is evident that the resonant mode that is sensitive to the refractive index, which appears at 900–1000 nm in Figure 4b, is absent in Figure A1a. Similarly, the resonant mode of the narrow linewidth in Figure 4c is not observed in Figure A1b. Thus, the reflectance spectra exhibit a definite polarization dependence.

In Figure A1, the spectral shapes are significantly different from those shown in Figure 4. The periodicity of 600 nm along the *x* axis results in a mode at 600 nm that hardly depends on the wavelength, as shown in Figure A1a. This type of mode is often referred to as Rayleigh anomaly. The modes appearing in Figure A1 are hardly dependent on the refractive index, whereas the modes at 600–1100 nm in Figure A1b depend on the refractive index. The only structural difference is the width of the Si bars: 540 nm and 300 nm in Figure A1a,b, respectively.

## References

[1] Raether H. (1988). Surface Plasmons on Smooth and Rough Surfaces and on Gratings.

[2] Myszka D.G., He X., Dembo M., Morton T.A., Goldstein B. (1998). Extending the Range of Rate Constants Available from BIACORE: Interpreting Mass Transport-Influenced Binding Data. Biophys. J..

[3] Breault-Turcot J., Poirier-Richard H.P., Couture M., Pelechacz D., Masson J.F. (2015). Single chip SPR and fluorescent ELISA assay of prostate specific antigen. Lab Chip.

[4] Joshi G.K., Deitz-McElyea S., Liyanage T., Lawrence K., Mali S., Sardar R., Korc M. (2015). Label-Free Nanoplasmonic-Based Short Noncoding RNA Sensing at Attomolar Concentrations Allows for Quantitative and Highly Specific Assay of MicroRNA-10b in Biological Fluids and Circulating Exosomes. ACS Nano.

[5] Yavas O., Svedendahl M., Dobosz P., Sanz V., Quidant R. (2017). On-a-chip Biosensing Based on All-Dielectric Nanoresonators. Nano Lett..

[6] Špačková B., Lynn N.S., Slabý J., Šípová H., Homola J. (2018). A Route to Superior Performance of a Nanoplasmonic Biosensor: Consideration of Both Photonic and Mass Transport Aspects. ACS Photonics.

[7] Zhou J., Tao F., Zhu J., Lin S., Wang Z., Wang X., Ou J.Y., Li Y., Liu Q.H. (2019). Portable tumor biosensing of serum by plasmonic biochips in combination with nanoimprint and microfluid. Nanophotonics.

[8] Zhu J., Wang Z., Lin S., Jiang S., Liu X., Guo S. (2020). Low-cost flexible plasmonic nanobump metasurfaces for label-free sensing of serum tumor marker. Biosens. Bioelectron..

[9] Miti A., Thamm S., Muller P., Csaki A., Fritzsche W., Zuccheri G. (2020). A miRNA biosensor based on localized surface plasmon resonance enhanced by surface-bound hybridization chain reaction. Biosens. Bioelectron..

[10] Bagra B., Mabe T., Tukur F., Wei J. (2020). A plasmonic nanoledge array sensor for detection of anti-insulin antibodies of type 1 diabetes biomarker. Nanotechnology.

[11] Ndao A., Hsu L., Cai W., Ha J., Park J., Contractor R., Lo Y., Kanté B. (2020). Differentiating and quantifying exosome secretion from a single cell using quasi-bound states in the continuum. Nanophotonics.

[12] Hsiao H.H., Hsu Y.C., Liu A.Y., Hsieh J.C., Lin Y.H. (2022). Ultrasensitive Refractive Index Sensing Based on the Quasi-Bound States in the Continuum of All-Dielectric Metasurfaces. Adv. Opt. Mater..

[13] Watanabe K., Iwanaga M. (2023). Nanogap enhancement of the refractometric sensitivity at quasi-bound states in the continuum in all-dielectric metasurfaces. Nanophotonics.

[14] Mollah M.A., Razzak S.A., Paul A.K., Hasan M.R. (2019). Microstructure optical fiber based plasmonic refractive index sensor. Sens. Bio-Sens. Res..

[15] Shaimerdenova M., Ayupova T., Sypabekova M., Tosi D. (2020). Fiber Optic Refractive Index Sensors Based on a Ball Resonator and Optical Backscatter Interrogation. Sensors.

[16] Wang Y., Gao R., Xin X. (2021). Hollow-core fiber refractive index sensor with high sensitivity and large dynamic range based on a multiple mode transmission mechanism. Opt. Express.

[17] Jain S., Choudhary K., Kumar S. (2022). Photonic crystal fiber-based SPR sensor for broad range of refractive index sensing applications. Opt. Fiber Technol..

[18] Ujah E., Lai M., Slaughter G. (2023). Ultrasensitive tapered optical fiber refractive index glucose sensor. Sci. Rep..

[19] Callewaert F., Velev V., Jiang S., Sahakian A.V., Kumar P., Aydin K. (2018). Inverse-designed stretchable metalens with tunable focal distance. Appl. Phys. Lett..

[20] Meem M., Banerji S., Pies C., Oberbiermann T., Majumder A., Sensale-Rodriguez B., Menon R. (2020). Large-area, high-numerical-aperture multi-level diffractive lens via inverse design. Optica.

[21] Tseng E., Colburn S., Whitehead J., Huang L., Baek S.H., Majumdar A., Heide F. (2021). Neural nano-optics for high-quality thin lens imaging. Nat. Commun..

[22] Piggott A.Y., Lu J., Lagoudakis K.G., Petykiewicz J., Babinec T.M., Vučković J. (2015). Inverse design and demonstration of a compact and broadband on-chip wavelength demultiplexer. Nat. Photonics.

[23] Hammond A.M., Oskooi A., Chen M., Lin Z., Johnson S.G., Ralph S.E. (2022). High-performance hybrid time/frequency-domain topology optimization for large-scale photonics inverse design. Opt. Express.

[24] Ong J.R., Chu H.S., Chen V.H., Zhu A.Y., Genevet P. (2017). Freestanding dielectric nanohole array metasurface for mid-infrared wavelength applications. Opt. Lett..

[25] Liu Z., Zhu D., Rodrigues S.P., Lee K.T., Cai W. (2018). Generative Model for the Inverse Design of Metasurfaces. Nano Lett..

[26] Iwanaga M. (2020). Non-Empirical Large-Scale Search for Optical Metasurfaces. Nanomaterials.

[27] Shen L., Ye Z., He S. (2003). Design of two-dimensional photonic crystals with large absolute band gaps using a genetic algorithm. Phys. Rev. B.

[28] Chen Y., Yu R., Li W., Nohadani O., Haas S., Levi A.F.J. (2003). Adaptive design of nanoscale dielectric structures for photonics. J. Appl. Phys..

[29] Goh J., Fushman I., Englund D., Vučković J. (2007). Genetic optimization of photonic bandgap structures. Opt. Express.

[30] Chen P.Y., Chen C.H., Wang H., Tsai J.H., Ni W.X. (2008). Synthesis design of artificial magnetic metamaterials using a genetic algorithm. Opt. Express.

[31] Iwanaga M. (2009). Optically deep asymmetric one-dimensional metallic grooves: Genetic algorithm approach. J. Opt. Soc. Am. B.

[32] Campbell S.D., Sell D., Jenkins R.P., Whiting E.B., Fan J.A., Werner D.H. (2019). Review of numerical optimization techniques for meta-device design. Opt. Mater. Express.

[33] Li L. (1997). New formulation of the Fourier modal method for crossed surface-relief gratings. J. Opt. Soc. Am. A.

[34] Li L. (1996). Formulation and comparison of two recursive matrix algorithm for modeling layered diffraction gratings. J. Opt. Soc. Am. A.

[35] Iwanaga M. (2016). Plasmonic Resonators: Fundamentals, Advances, and Applications.

[36] Rakić A.D., Djurušić A.B., Elazar J.M., Majewski M.L. (1998). Optical properties of metallic films for vertical-cavity optoelectronic devices. Appl. Opt..

[37] Palik E.D. (1991). Handbook of Optical Constants of Solids II.

[38] Sakoda K. (2005). Optical Properties of Photonic Crystals.

[39] Ochiai T., Sakoda K. (2001). Dispersion relation and optical transmittance of a hexagonal photonic crystal slab. Phys. Rev. B.

[40] Iwanaga M. (2018). All-Dielectric Metasurfaces with High-Fluorescence-Enhancing Capability. Appl. Sci..

[41] Iwanaga M. (2020). All-Dielectric Metasurface Fluorescence Biosensors for High-Sensitivity Antibody/Antigen Detection. ACS Nano.

[42] Dong Z., Gorelik S., Paniagua-Dominguez R., Yik J., Ho J., Tjiptoharsono F., Lassalle E., Rezaei S.D., Neo D.C.J., Bai P. (2021). Silicon Nanoantenna Mix Arrays for a Trifecta of Quantum Emitter Enhancements. Nano Lett..

[43] Iwanaga M., Hironaka T., Ikeda N., Sugasawa T., Takekoshi K. (2023). Metasurface Biosensors Enabling Single-Molecule Sensing of Cell-Free DNA. Nano Lett..

[44] Gomez-Medina R., Garcia-Camara B., Suarez-Lacalle I., González F., Moreno F., Nieto-Vesperinas M., Saenz J.J. (2011). Electric and magnetic dipolar response of germanium nanospheres: Interference effects, scattering anisotropy, and optical forces. J. Nanophotonics.

[45] Kuznetsov A.I., Miroshnichenko A.E., Brongersma M.L., Kivshar Y.S., Luk’yanchuk B. (2017). Optically resonant dielectric nanostructures. Science.

[46] He L., Yi Y., Zhang J., Xu X., Tang B., Li G., Zeng L., Chen J., Sun T., Yi Z. (2024). A four-narrowband terahertz tunable absorber with perfect absorption and high sensitivity. Mater. Res. Bull..

[47] Homola J., Koudela I., Yee S.S. (1999). Surface plasmon resonance sensors based on diffraction gratings and prism couplers: Sensitivity comparison. Sens. Actuator B-Chem..

